# Increased Innate Lymphoid Cells in Periodontal Tissue of the Murine Model of Periodontitis: The Role of AMP-Activated Protein Kinase and Relevance for the Human Condition

**DOI:** 10.3389/fimmu.2017.00922

**Published:** 2017-08-15

**Authors:** Xu Qin, Md Nasrul Hoda, Cristiano Susin, Julie N. Wheeler, Brendan Marshall, Libby Perry, Nancy Saad, Lin Yin, Ranya Elsayed, Mohammed Elsalanty, Rafik Abdelsayed, Jack C. Yu, Krishnan M. Dhandapani, Omid Akbari, Mahmood S. Mozaffari, Babak Baban

**Affiliations:** ^1^Huazhong University of Science and Technology, Wuhan, China; ^2^Augusta University, Augusta, GA, United States; ^3^Grovetown Family Dental, Grovetown, GA, United States; ^4^University of Southern California, Los Angeles, CA, United States

**Keywords:** periodontitis, innate lymphoid cells, innate lymphoid cells, AMP-activated protein kinase, inflammation, cytokines, human

## Abstract

Innate lymphoid cells (ILCs) are master regulators of immune and inflammatory responses, but their own regulatory mechanisms and functional roles of their subtypes (i.e., ILC1s–ILC3s) remain largely unresolved. Interestingly, AMP-activated protein kinase (AMPK), influences inflammatory responses, but its role in modulation of ILCs is not known. Periodontitis is a prevalent disorder with impairment of immune and inflammatory responses contributing importantly to its pathogenesis; however, neither the role of ILCs nor AMPK has been explored in this condition. We tested the hypotheses that (a) periodontitis increases ILCs and expression of relevant cytokines thereby contributing to inflammation and (b) knockdown of AMPK worsens indices of periodontitis in association with further increases in subtypes of ILCs and cytokine expression. The studies utilized wild-type (WT) and AMPK knockout (KO) mice, subjected to ligature-induced periodontitis or sham operation, in association with the use of micro-CT for assessment of bone loss, immunogold electron microscopy to show presence of ILCs in periodontal tissues, flow cytometry for quantitative assessment of subtypes of ILCs and RT-polymerase chain reaction analyses to measure mRNA expression of several relevant cytokines. The results for the first time show (a) presence of each subtype of ILCs in periodontal tissues of sham control and periodontitis animals, (b) that periodontitis is associated with increased frequencies of ILC1s–ILC3s with the effect more marked for ILC2s and differential phenotypic marker expression for ILC3s, (c) that AMPK KO mice display exacerbation of indices of periodontitis in association with further increases in the frequency of subtypes of ILCs with persistence of ILC2s effect, and (d) that periodontitis increased mRNA for interleukin (IL)-33, but not IL-5 or IL-13, in WT mice but expression of these cytokines was markedly increased in AMPK KO mice with periodontitis. Subsequently, we showed that human periodontitis is associated with increases in each ILCs subtype with the effect more marked for ILC2s and that mRNA expressions for IL-33 and IL-5 are markedly greater for sites affected by periodontitis than healthy sites. Collectively, these novel observations indicate a pivotal role for ILCs in pathogenesis of periodontitis and that AMPK is a regulator of their phenotype expression in this condition.

## Introduction

Periodontal diseases are a group of diseases that affect tissues that support and anchor the teeth. Periodontitis is the most advanced form of the periodontal diseases, progresses relatively slowly in most people, and is typically more evident in adulthood (1, 2). According to the Center for Disease Control, periodontitis affects over 60 million Americans, contributing significantly not only to the burden of oral diseases, but likely to a number of systemic diseases including diabetes mellitus and cardiovascular disorders (3).

The human microbiome project has revealed that the oral environment is likely populated by about 1,200 bacteria, about 350 of which are implicated in pathogenesis of periodontitis (4, 5). In terms of pathogenesis, periodontitis is initiated by dysbiotic bacterial communities forming biofilms. The initial response to bacterial infection is a local inflammatory reaction that activates the innate immune system. Amplification of this initial localized response results in the release of an array of cytokines and other mediators, with propagation of inflammation through the periodontal tissues (6, 7). The failure to contain this “inflammatory front” within gingival tissue results in expansion of the response adjacent to alveolar bone. This inflammatory process then drives the destruction of connective tissue and alveolar bone that is the cardinal sign of periodontitis (1, 2, 6). Therefore, it is imperative to explore mechanisms responsible for the inflammatory and immune responses in the pathogenesis of periodontitis.

Recent studies have identified a new group of innate lymphocytes collectively called innate lymphoid cells (ILCs), located in barrier surfaces of the skin, airways, and intestine (8–13). ILCs are believed to have dual function including initiation of innate immunity and establishing homeostatic balance in tissues *via* inflammation and tissue repair. Under normal conditions, ILCs play an important role in maintaining tissue integrity; during the course of inflammation and pathological conditions, they participate in pathogen clearance, lymphoid organogenesis, and tissue remodeling. Although ILCs are part of the innate immune system, they can be positioned at the interface between acquired immune cells and myeloid cells in a wide variety of mucosal and epithelial compartments (8–13).

Innate lymphoid cells are divided into three classes. The development of all classes of ILCs require cytokine signaling as well as transcription factor Id2. Class 1 consists of natural killer cells and IFNγ-producing ILCs that utilize T-bet (transcription factor) for lineage commitment. Class 2 consists of type 2 cytokine-producing ILCs [e.g., interleukin (IL)-5, IL-13], which express the transcription factor GATA-3 and depend on transcription factor RORα for their activity and development. The third class of ILCs constitutes pro-inflammatory cytokines IL-17 and/or IL-22 producing ILCs that require transcription factor RORγt for their lineage characteristics and function. Several studies have proposed multiple immune regulatory roles for ILCs in different tissues and during inflammatory responses (13, 14). Despite their important roles in immune responses against pathogens and in the maintenance of tissue homeostasis, uncontrolled activation of ILCs may elicit inflammatory responses that further result in pathological conditions. Since periodontitis is a prevalent inflammatory disease in which cytokines and immune responses play essential roles, it is plausible to suggest pathogenic role for ILCs in this condition. Importantly, while ILCs are increasing recognized as master regulators of immune and inflammatory responses, a set of transcription factors regulates ILCs development and concedes specific functions to different subtypes of ILCs. The advent of AMP-activated protein kinase (AMPK) knockout (KO) mice provides a unique opportunity to explore the impact of AMPK on subtypes of ILCs (15). This investigation is warranted because while the role of AMPK in cellular bioenergetics is well established, its role in regulation of immune and inflammatory responses is increasing being recognized (16, 17). However, the role of AMPK in either periodontitis or its impact on ILCs is not established. Thus, utilizing the murine model of ligature-induced periodontitis (18), we tested the hypotheses that (a) periodontitis is associated with both upregulation of and differential impact on subtypes of ILCs and expression of relevant cytokines and (b) knockdown of AMPK worsens indices of periodontitis in association with further increases in subtypes of ILCs and cytokine expression. The results clearly established a role for both ILCs and AMPK in the murine model of periodontitis. Thus, subsequent studies explored the relevance of these findings for the human condition utilizing periodontal tissues obtained from healthy sites and those with severe periodontitis.

## Materials and Methods

### Preclinical Studies

#### Animals

Wild-type (WT) (BALB/c) and α1 AMPK KO mice of similar background of 9–10 weeks of age were used these studies. The generation of α1 AMPK KO mice and their genotyping have been described previously (15); for this study, effectiveness of α1 AMPK deletion was confirmed by performing polymerase chain reaction (PCR) on tail tissue and flow cytometry analysis of peripheral blood. The use of animals for this study conformed to guidelines of Institutional Animal Care and Use Committee of Augusta University. The animals were housed under identical standard conditions with free access to food and tap water.

### Ligature-Induced Periodontitis

#### Placement of Ligatures

**Figure 1:**
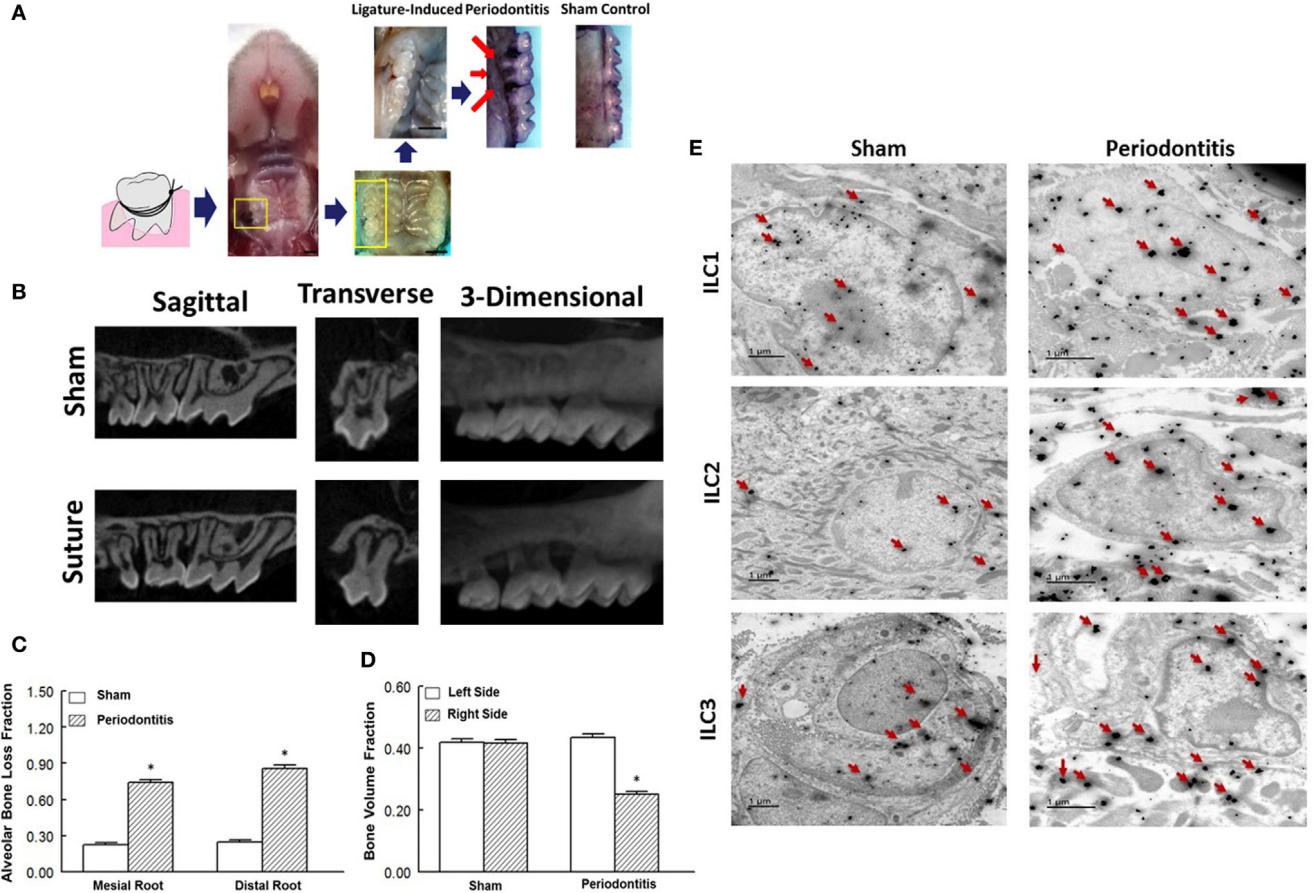
Ligature-induced periodontitis model in mice and presence of innate lymphoid cells (ILCs) in periodontal tissues. **(A)** Gross view of ligature-induced periodontitis, second molar, after 7 days of suture placement; arrows point to the suture being covered by palatal tissue and consequent bone loss around the ligated tooth; scale bar = 1 mm. **(B)** Sagittal, transverse, and 3-D micro-CT images of second molar and associated structures. Micro-CT images show marked bone loss in response to placement of suture compared to sham operation. Bar graphs show alveolar bone loss fraction around mesial and distal roots **(C)** and bone volume fraction on right and left side second-molar teeth **(D)** in experimental groups. **(E)** Transmission electron microscopy images of periodontal tissues obtained from sham-operated control and ligature-induced periodontitis mice. Each panel shows one cell displaying two-sized gold particles; large particles (red arrows) confirming specific markers (intracellular and nuclear/perinuclear stains) identifying ILC1s, ILC2s, and ILC3s. *p < 0.05 compared to the other condition/group.

Prior to ligature placement, all mice were anesthetized *via* intraperitoneal injections of a mixture of ketamine (120 mg/kg) and xylazine (16 mg/kg). To induce periodontitis, utilizing a microscope, a 5-0 silk ligature (Roboz Surgical Instrument Co., MD, USA) was carefully placed around the cervical margin of the right maxillary second molar and knotted mesiobuccally (Figure 1). The contralateral molar in each mouse was left unligated to serve as the baseline control for periodontal bone evaluation (18). After 7 days, under anesthesia, ligatures were gently removed. After obtaining periodontal tissues for flow cytometry-based analyses (below), skulls were collected and prepared for micro-CT analyses.

### Clinical Studies

#### Sample Collection

Periodontal tissue samples (*n* = 10 subjects) were obtained from patients admitted to the Clinic of Periodontics, Dental College of Georgia at the Augusta University (Augusta, GA, USA) and from patients at Grovetown Family Dental (Dr. Wheeler’s Clinic, Grovetown, GA, USA). These patients had generalized and severe chronic periodontitis as indexed by bone loss ≥50%, probing pocket depth ≥7 mm, and bleeding on probing ≥4 teeth. Tissues from unaffected areas were included as control samples. The institutional human review board approved the study protocol. None of the subjects had a known systemic disorder that could have affected the periodontal condition.

### Micro-CT Detection of Murine Periodontitis

Three maxillary blocks in each group were processed for SkyScan 1174 (Micro Photonics Inc., Allentown, PA, USA), a compact X-ray micro-CT scanner for periodontal bone assessment. Scanning was performed with a rotational angle of 360° around the longitudinal axis of the second molar tooth, utilizing a spatial resolution of 15.9 µm at 50 kV, 800 µA, and a 0.5° rotation step with an exposure time of 2,000 ms (19, 20).

Periodontal bone heights were measured as the distances from the cementoenamel junction (CEJ) to the alveolar bone crest (ABC) in the interdental region between the first and second molars (M1–M2) or the second and third molars (M2–M3). The distances of the CEJ and the root apex (RA) appearing in the micro-CT slice were also calculated. The ratios of CEJ-ABC and CEJ-RA were taken to assess the percentage of vertical bone remaining.

Volumetric measurements [i.e., bone volume fraction (BVF)] were carried out within the region of interest (ROI). The ROI of volumetric analysis of the maxillary second tooth-supporting alveolar bone included the following landmarks: the roof of the furcation, the RA of the second molar, the mesial roots of the third molar, and the distal roots of the first molar. The ROIs were drawn by two different examiners to ensure repeatability according to morphological landmarks.

For image reconstruction, 2-D virtual sections of maxillary second molar teeth were acquired in the coronal, axial, and sagittal planes by the Skyscan CT-analyzer (CTAn) software. The CTAn software was further employed for 3-D analysis and quantification of the volume of interfacial gaps and voids within the ROI. The Skyscan CT-Volume software was used for 3-D visualization of periodontal bone around the second molar (19, 20).

### Sample Processing and Preparation

Obtained periodontal tissues (from human and mice) were divided into two parts. One part was weighed and processed for flow cytometry analysis while the other part was maintained and later processed for RT-PCR as described below. For flow cytometry analysis, tissues were placed in a tissue culture dish with 1 ml PBS + 2% FCS, 2 mg/ml of Collagenase Type II, and 1 mg/ml of DNAse Type I for 30 min at 37°C. Thereafter, samples were sieved through a cell strainer (BD Biosciences, San Diego, CA, USA), followed by centrifugation (1,500 rpm, 5 min) to prepare single-cell suspensions for flow cytometry analysis.

### Electron Microscopy

The protocol for electronic microscopic assessment of ILCs subtypes was recently described in detail by our group (21). Briefly, we utilized double immunogold labeling with silver enhancement for transmission electron microscopy. Following tissue fixation and processing, thin sections of 75 nm thickness were cut on a Leica UC6 Ultramicrotome (Leica Microsystems, Inc.) and collected on 200-mesh nickel grids. Sections were etched in 2% H_2_O_2_ in PBS for 20 min and washed three times for 5 min with PBS followed by quenching in 1-M ammonium chloride in PBS and washed three times for 5 min with PBS. Thereafter, sections were blocked in 0.4% BSA in PBS for 2–4 h then placed in the first-primary antibody solution overnight at 4°C. Sections were then rinsed in PBS three times for 10 min followed by treatment with anti-primary species specific Nanogold (1:1,000) (Nanoprobes, Inc.) in 0.4% BSA in PBS for 2 h at room temperature; this was followed by washing three times for 10 min in PBS, two times for 10 min in deionized water, then silver enhanced for 12 min in HQ Silver (Nanoprobes, Inc.). This enhancement results in large gold particles labeling the first-primary antibody. Sections were then washed in ice cold H_2_O 3°, 5 min, to stop enhancement. Thereafter, sections were placed in a second-primary antibody, and the steps above for the first-primary antibody were followed for the secondary nanogold antibody until the second enhancement step. The sections were then enhanced with HQ Silver for only 6 min, resulting in small gold particle labeling of the second-primary antibody. Sections were then washed three times for 5 min with deionized water and stained 5 min with 2% uranyl acetate. Grids were allowed to dry, and tissue was observed in a JEM 1230 Transmission Electron Microscope (JEOL USA Inc., Peabody, MA, USA) at 110 kV and imaged with an UltraScan 4000 CCD camera and first light digital camera controller (Gatan Inc., Pleasanton, CA, USA).

### Preparative and Analytical Flow Cytometry

Preparative cell sorts were performed on cells stained with fluorochrome-conjugated mAbs (sources as detailed, below) using a Mo-Flo 4-way flow cytometer (DakoCytomation) equipped with 488 nm argon (for FITC, PE, PE-CY5) and 647 nm krypton (for allophycocyanin) lasers. Cells were gated based on forward and side scatter properties and on marker combinations to select cells of interest (21–24). Total ILCs were gated as Lin^−^CD127^+^ lymphocytes. The lineage cocktail of antibodies included FITC-conjugated anti-CD3, anti-CD4, anti-CD14, anti-CD16, anti-CD19, anti-CD8, anti-CD15, anti-CD20, anti-CD33, anti-CD34, anti-CD203 (eBioscience), and anti-FcεRI (BioLegend) with PerCP-conjugated CD127 (BioLegend). Subsequently, group 1 ILCs were identified as CRTH2^−^cKit^−^CD56^−^ cells positive for T-bet, group 2 ILCs as CRTH2^+^cKit^+/−^ cells positive for GATA-3, and group 3 ILCs as CRTH2^−^cKit^+^ cells positive for RORγt (CRTH2 from abcam and cKit, CD56 from eBioscience). To detect periodontitis-related changes in phenotypic marker expression for each subset of ILCs, we carried out *in vitro* studies as follow. Periodontal cells were obtained from WT mice without or with periodontitis (*n* = 3/group). Thereafter, each subset of ILCs was identified, as described earlier, followed by their incubation (4 h) either in the absence or presence of cytokines: ILC1s: IL-12, IL-15, and IL-18; ILC2s: IL-33, IL-25, thymic stromal lymphopoietin; ILC3s: IL-1β and IL-23 (all purchased from BioLegend). This was followed by flow cytometry-based analysis for expression of phenotypic markers as follow: ILC1s: CD127 and CD49a; ILC2s: ST2 (IL33Rα) and killer cell lectin-like receptor G1; ILC3s: NKp46.

To test functionality of ILCs, we focused on cytokine expression by ILC2s because they showed the more robust change under our experimental conditions. Thus, we stimulated total ILCs with 1 mg/ml PMA plus 0.5 mg/ml ionomycin in the presence of Brefeldin A (Sigma-Aldrich) for 3 h and stained them for intracellular cytokine production. Briefly, cells were harvested and fixed/permeabilized using the BD Cytofix/Cytoperm™ Plus solution kit (BD Biosciences). After 20 min of incubation, cells were washed by adding 2 ml of PBS (containing 0.1% triton or other permeabilizing detergent), centrifuged at 1,500 rpm for 5 min, the supernatant was discarded, and the pellet was resuspended in the remaining volume. Corresponding antibodies against intracellular cytokines were prepared in a permeabilization buffer. Following incubation, all stained cells were washed and resuspended in 400 µl of flow cytometry staining buffer and analyzed using CellQuest software through four-color Calibur flow cytometer (BD Biosciences). As a gating strategy, isotype-matched controls were analyzed in each sample to set the appropriate gates; representative data are reported in relevant figures. For each marker, samples were analyzed with duplicate measurements. To minimize false-positive events, the number of double-positive events detected with the isotype controls was subtracted from the number of double-positive cells stained with corresponding antibodies (not isotype control), respectively.

### RNA Extraction and Real-time Quantitative PCR

RNA was extracted using Trizol reagent (Invitrogen, CA, USA) according to the manufacturer’s protocol. Total RNA was treated with RNase-free DNase (Epi-centre, Madison, USA) and then reverse transcribed to create cDNA. The resulting transcripts were then quantified by the real-time quantitative PCR on a StepOneplus real-time DNA amplification system (Applied Biosystems, Foster City, CA, USA) utilizing the SYBR Green PCR protocol with RT SYBR^®^ Green qPCR Master Mixes (PA-112, SAbiosciences, Qiagen) on an Applied Biosystems 7900HT Sequence Detection System (Applied Biosystems, USA). Predesigned primers specific for amplification of IL-33, IL-5, and IL-13 were applied. For each sample, transcript quantity was normalized to the amount of beta-actin expression. Amplification was carried out in a total volume of 20 µl for 40 cycles of 30 s at 95°C and 30 s at 60°C (25, 26).

### Statistics

Data were analyzed using Student’s *t*-test (human data) and the analysis of variance followed by Newman–Keuls *post hoc* test to establish significance (*p* < 0.05) among groups for all other data. Data are reported as mean ± SEM.

## Results

### Animal Model of Periodontitis and Demonstration of Presence of ILCs in Periodontal Tissue

We initially explored the efficacy of ligature placement around the second molar for induction of periodontitis in WT mice (i.e., BALB/c); non-ligature contralateral second molar served as sham control. The ligated sites showed abrupt acceleration of bone loss as revealed by a marked effect 7-day post-ligation (Figure 1A). At this time, abnormal fenestration was noted on the buccal side that subsequently led to the collapse of overlying alveolar bone. To assess the extent of bone loss, utilizing micro-CT, the CEJ-ABC distances were measured at predetermined sites on buccal or palatal surfaces of the ligated tooth and of the non-ligated corresponding contralateral side (sham control). Relative changes in bone heights were calculated for each ligated site by subtracting its CEJ-ABC distance value from that of the corresponding non-ligated site and data are shown for mesial and distal roots of the second molar (Figure 1B). As shown in Figure 1C, the ligated molar tooth (i.e., periodontitis) displayed significant alveolar bone loss compared to the sham control tooth. Consistent with this finding, ligature-induced periodontitis was associated with significant decrease in BVF compared to the sham control side (Figure 1D). Importantly, using immunogold labeling, we observed the presence of each subtype of ILCs in periodontal tissues of both sham-operated control and those in whom periodontitis was induced by ligature placement. Each panel under Figure 1E shows one cell displaying two-sized gold particles; large particles (red arrows) confirm specific markers (intracellular and nuclear/perinuclear stains) identifying ILC1s, ILC2s, and ILC3s. Thus, the ligatured-induced model of periodontitis was used in subsequent studies.

### Effect of Knockdown of AMPK on Development of Ligature-Induced Periodontitis

**Figure 2:**
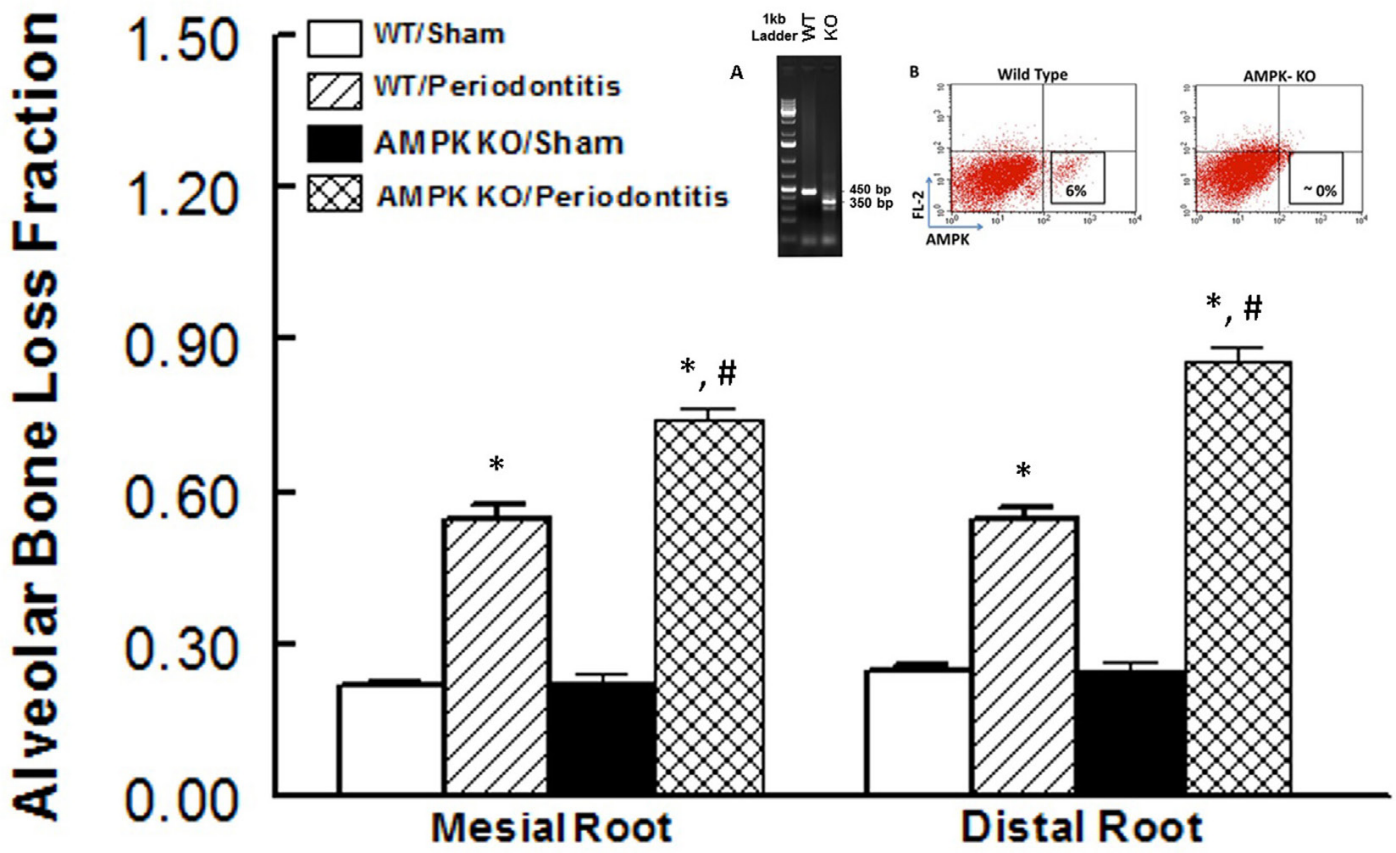
Effect of AMP-activated protein kinase (AMPK) knockdown on murine periodontitis model. Alveolar bone loss fraction around mesial and distal roots of the second molar in sham-operated and ligature-induced periodontitis in wild-type (WT) mice and AMP-activated protein kinase knockout (AMPK KO) mice. The inset shows successful genetic deletion of WT α1 AMPK as revealed by the absence of polymerase chain reaction band at 450 bp while the mutant band was detected at 350 bp [inset panel (A); tail tissue] and flow cytometry for AMPK protein expression [inset panel (B); peripheral blood] thereby confirming success of AMPK deletion in AMPK KO compared to WT mice. *p < 0.05 compared to their sham-operated counterparts. ^#^*p* < 0.05 compared to WT/periodontitis group.

Figure 2 shows alveolar bone loss fraction for WT and AMPK KO mice subjected to ligature-induced periodontitis or sham operation; Figure 2 inset shows effectiveness of AMPK deletion as determined by the absence of PCR band at 450 bp while the mutant band was detected at 350 bp (inset panel A) and flow cytometry analysis of peripheral blood for AMPK protein expression (inset panel B). As expected, ligature placement caused significant increase in bone loss on the mesial and distal roots of maxillary second molar tooth compared to sham-operated group. Interestingly, bone loss in ligature-placed AMPK KO mice was more marked compared to their WT counterparts, which achieved statistical significance.

### Effects of Periodontitis and AMPK KO on Periodontal Tissues

Inflammatory responses and metabolic regulation are highly integrated and their interaction a pivotal determinant of homeostasis; dysregulation of these processes can lead to a cluster of chronic metabolic and inflammatory disorders (16). Indeed, transition of immune cells from resting to active state requires and is associated with significant metabolic changes. Thus, the interface between inflammation and metabolic changes may be considered as a determinant of immune tolerance. Our inadequate understanding of the relationship between inflammation and metabolism in periodontitis prompted us to explore the impact of the disease on ILCs in the context of assessment of the role of AMPK; AMPK, an archetypal metabolic regulator, is a highly conserved protein kinase that exists in essentially all eukaryotic cells including those of immune system (16, 17).

**Figure 3:**
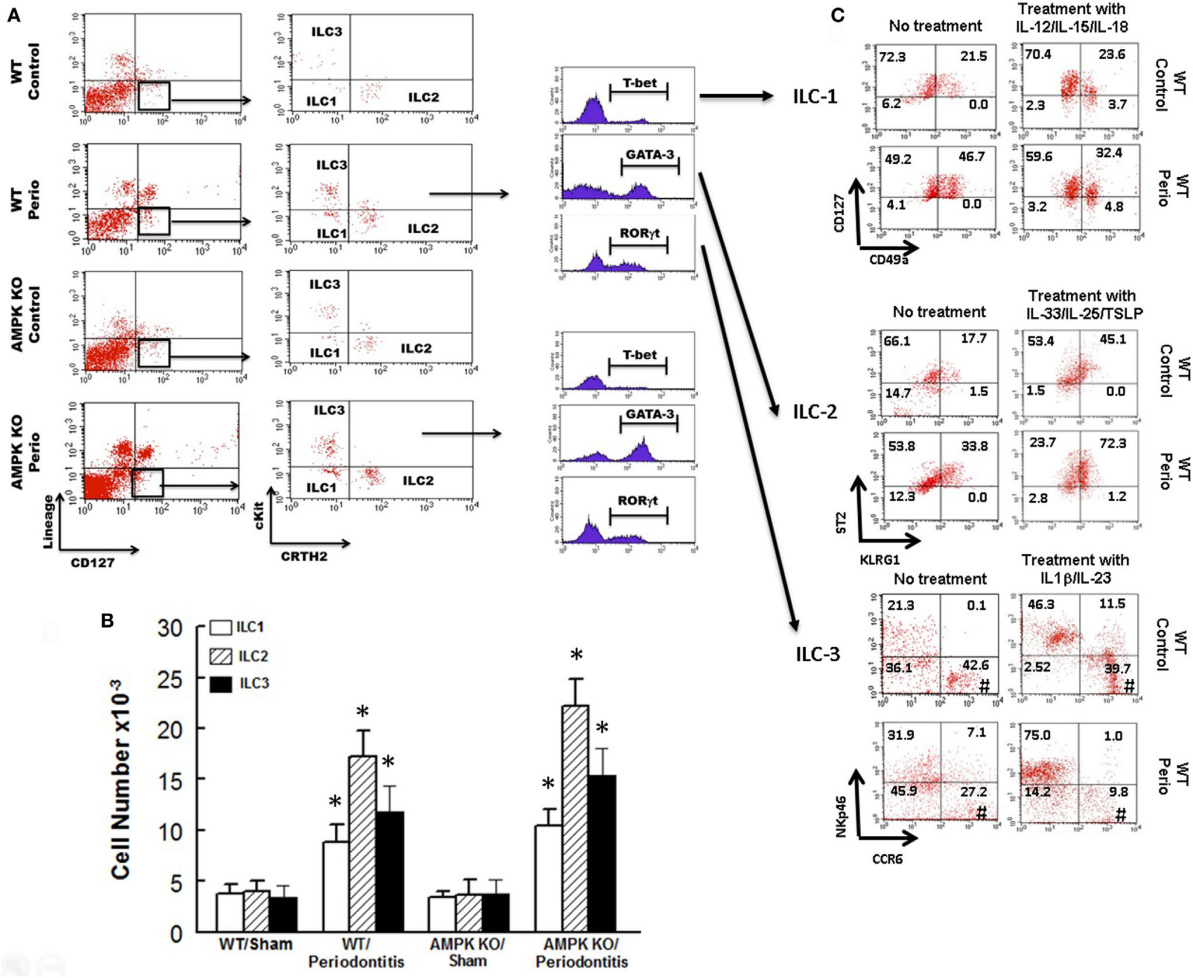
Innate lymphoid cells frequency and subtypes. Representative panels show multiparameter flow cytometry and gating strategy to determine subsets of innate lymphoid cells (ILCs) in experimental groups as described under Section “Materials and Methods.” Briefly, ILCs were initially identified as lineage negative CD127^+^ lymphocytes followed by analyses of signature transcription factor for each subset of ILCs as follow: ILC1s: T-bet; ILC2s: GATA-3, and ILC3s: RORγt **(A)**. **(B)** Average ± SEM of each ILCs’ subtype for sham-operated and ligature-induced periodontitis in wild-type (WT) and AMP-activated protein kinase knockout (AMPK KO) mice. **(C)** Further flow cytometry-based evaluation for expression of specific markers for each subset of ILCs as follow: ILC1s: CD127 and CD49a; ILC2s: ST2 and killer cell lectin-like receptor G1 (KLRG1); ILC3s: NKp46 and CCR6; the assessment was carried out without or with prior *in vitro* treatment with relevant cytokines as shown for each subset of ILCs as described under Section “Materials and Methods.” Value in each quadrant represents percent of total cells for each group/condition/treatment; ^#^quadrants with most noted changes between the two groups under each condition. *p < 0.05 compared to their sham-operated counterparts.

Figure 3 shows representative dot matrices for ILCs in experimental groups (Figure 3A); analysis of the different ILCs subsets was characterized by the combination of surface marker expression and of specific transcription factors as detailed above. All three classes of ILCs were detected in periodontal tissues of experimental groups. Both WT sham and AMPK KO sham groups showed similar frequencies of each ILCs subtypes (Figure 3B). Importantly, however, periodontitis caused marked increases in ILC1s–ILC3s subtypes in periodontal tissues of WT mice, but the effect was more marked for ILC2s compared to their WT/sham counterparts (Figure 3B). The AMPK KO mice subjected to ligature-induced periodontitis displayed a similar pattern to their WT counterparts, achieving higher levels albeit non-significant (Figure 3B).

In light of marked upregulation of ILCs in the murine model of periodontitis, we carried out further *in vitro* studies to determine whether the disease impacts phenotypic marker expression under baseline condition and in response to cytokine treatment as described under Section “Materials and Methods.” Figure 3C shows representative dot matrices, for each subset of ILCs, displaying percent of total cells in each quadrant for each condition/treatment. The most noted observation relates to phenotypic marker expression for ILC3s. Accordingly, ILC3s of periodontal tissues from control animals (i.e., without periodontitis) display more marked CCR6 expression either without or with IL-1β/IL-23 treatment compared to those of periodontitis model; however, the effect was more marked for cytokines-treated ILC3s (Figure 3C).

### Effects of Periodontitis and AMPK KO on Cytokine Expression

**Figure 4:**
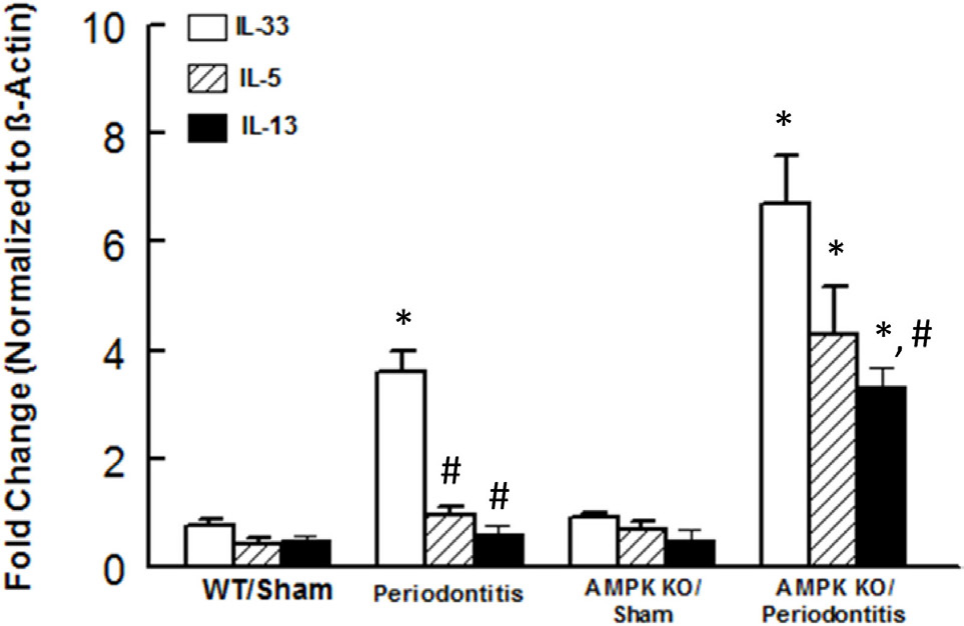
Cytokine expression in periodontal tissues of mice. Bar graphs show mRNA expression for cytokine expression in periodontal tissues obtained from sham-operated and ligature-induced periodontitis in wild-type (WT) and AMP-activated protein kinase knockout (AMPK KO) mice. *p < 0.05 compared to their sham-operated counterparts. ^#^*p* < 0.05 compared to interleukin (IL)-33 expression in the same group.

We next focused on assessment of functional status of ILC2s since this subset of ILCs showed most marked changes in response to periodontitis and AMPK knockdown. We assessed expression levels for IL-5 and IL-13 in periodontal tissues since these cytokines are prominently expressed by ILC2s; in our assessment, we also included IL-33 since it is an endogenous stimulator of cytokine expression by ILC2s. As shown in Figure 4, periodontal tissue expression of each cytokine was similar between sham-operated WT and AMPK KO mice. Induction of periodontitis in wild mice markedly increased expression of IL-33 but expression levels of IL-5 and IL-13 resembling those of sham-operated groups. Interestingly, however, induction of periodontitis in AMPK KO mice further increased IL-33 mRNA expression and also caused significant increases in expression levels of both IL-5 and IL-13.

### Status of ILCs and Cytokine Expression in Human Periodontitis

**Figure 5:**
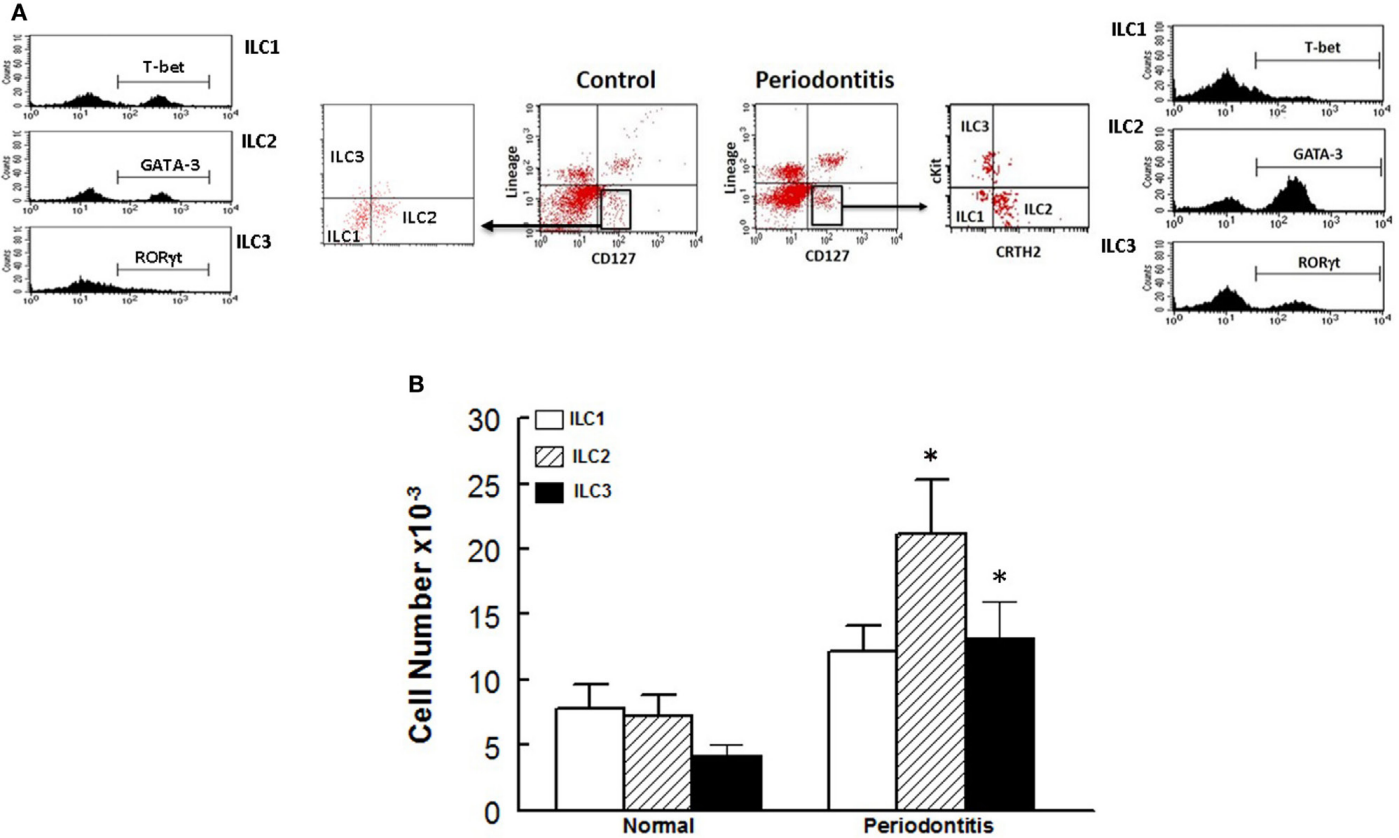
Innate lymphoid cells frequency and subtypes in human periodontitis. Representative panels show multiparameter flow cytometry gating strategy to determine subsets of innate lymphoid cells (ILCs) in experimental groups as described under Section “Materials and Methods.” Briefly, ILCs were initially identified as lineage negative CD127^+^ lymphocytes followed by analyses for signature molecules for each subset of ILCs as follow: ILC1: T-bet; ILC2: GATA-3 and ILC3: RORγt **(A)**. **(B)** Average ± SEM of each ILCs subtype for periodontal tissues obtained from healthy sites and those affected by periodontitis of human subjects. *p < 0.05 compared to healthy control sites.

**Figure 6:**
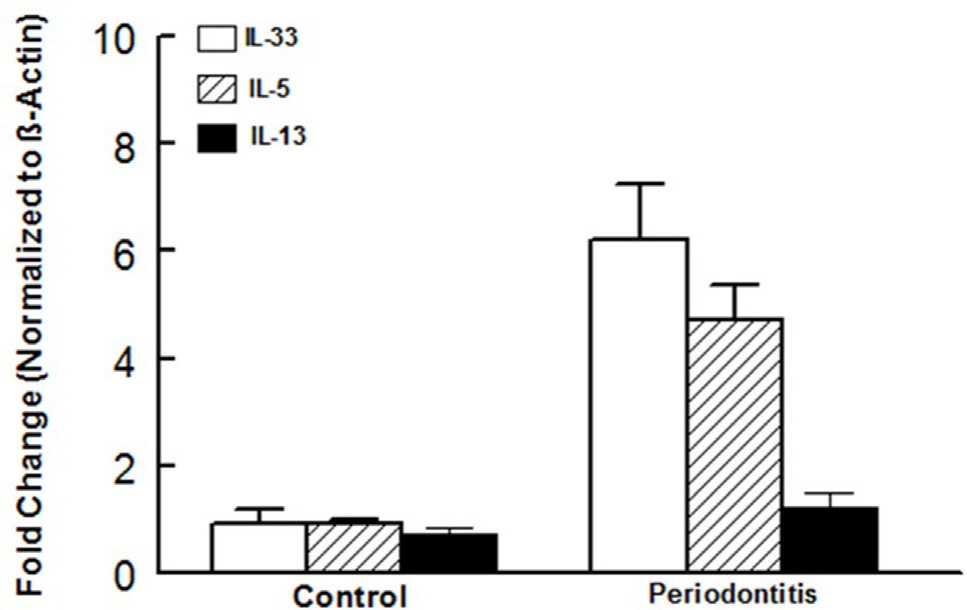
Cytokine expression in human periodontal tissues. Bar graphs show mRNA expression for cytokine expression in periodontal tissues obtained from healthy sites and those affected by periodontitis of human subjects. *p < 0.05 compared to same cytokine in the control group. ^#^*p* < 0.05 compared to interleukin (IL)-33 or IL-5 expression in the same group.

In light of our observations with the murine model of ligature-induced periodontitis, we explored whether similar changes in ILCs and cytokine expression are associated with human periodontitis. As shown in Figure 5, periodontal tissues from sites clinically established to be affected by severe periodontitis displayed increases in each subset of ILCs with the effect more marked for ILC2s and ILC3s compared to periodontal tissues obtained from healthy sites. Indeed, the pattern of changes in ILCs was remarkably similar between human periodontitis and the murine model of ligature-induced periodontitis (Figure 3). Figure 6 shows that message expression was significantly increased for IL-33 and IL-5, but not IL-13, for periodontal tissues obtained from sites affected by periodontitis compared to those obtained from healthy sites.

## Discussion

The present study shows that ILCs are present in periodontal tissues of mice. Further, periodontal tissues of the ligature-induced murine model of periodontitis display (a) significant increases in each subset of ILCs, an effect more marked for ILC2s and those with knockdown of AMPK and (b) increased expression of IL-33 but knockdown of AMPK is associated with significant increases in expression of ILC2s-generated cytokine: IL-33, IL-5, and IL-13. Importantly, periodontal tissues of human subjects obtained from sites affected by periodontitis show remarkable similarities to those of the murine model of periodontitis as revealed by increases in each subset of ILCs with the effect more marked for ILC2s and increased expression of mRNA for IL-33 and IL-5. Collectively, these novel observations indicate important pathogenic roles of ILCs in periodontitis and also suggest that AMPK is a modulator of ILCs frequency and function in this condition.

Innate lymphoid cells are found in many organs in mice and human subjects. Increasing evidence suggests a crucial role for ILCs in initiation, modulation, and resolution of inflammatory responses (8–14). ILCs have lymphoid lineage because they are derived from the common lymphoid progenitor but lack antigen-specific receptors and other lineage markers thereby distinguishing them from conventional B and T lymphocytes. Interestingly, however, functional specialization of ILCs and their developmental programs resemble those of CD4^+^ T helper (Th) cell subsets. As a result, ILC1s, ILC2s, and ILC3s are increasingly considered as the innate counterparts of Th-1, Th-2, and Th-17 cells. In terms of function, ILC1s generate INF-γ and TNF-α while ILC2s cytokines include IL-5 and IL-13 and ILC3s produce IL-22 and/or IL-17 among others (8–14, 27). Importantly, however, regulation of frequency and function of ILCs remain to be better established although Janus Kinase/signal transducer and activator of transcription family of proteins are believed to be important in ILCs biology and some of their effector functions (27, 28). In this context, it is noteworthy that while the role of AMPK in regulation of cellular metabolism and bioenergetics is well-recognized, more recent studies suggest an important role for AMPK in regulation of immune and inflammatory responses (15–17). Indeed, processes involved in activation of macrophages and Th-17 cells include a shift in cellular metabolism toward enhanced glucose uptake, glycolysis, and increased activity of pentose phosphate pathway. Consequently, the role of AMPK in regulation of immunity and inflammation, *via* mechanisms dependent on metabolism of immune and inflammatory cells, is offering enticing prospects of novel therapeutic modalities for inflammatory diseases (17). Importantly, however, neither the role of ILCs nor their regulation/modulation via AMPK in periodontitis have been explored previously but are the subject of our current report.

For our studies, we used the ligature-induced murine model of periodontitis. Accordingly, we initially established that placement of a ligature around the maxillary molar tooth causes a relatively rapid and significant reduction in alveolar bone as revealed by marked increases in fractional alveolar bone loss around mesial and distal roots coupled with significant reduction in bone volume as measured by micro-CT. Having established the efficacy of ligature placement, subsequent experiments utilized WT and AMPK KO mice which were subjected to ligature-induced periodontitis or sham operation to more fully examine the development of periodontitis in the context of assessment of ILCs and relevant cytokines. The results indicate that knockdown of AMPK is associated with exacerbated bone loss thereby implicating a role for AMPK in pathogenesis of periodontitis. To our knowledge, this is the first report that has directly explored the impact AMPK on periodontitis. In the context of the role of AMPK in periodontitis, a recent study has examined the effects of melinjo resveratrol on ligature-induced periodontitis in rat (29). Authors conclude that the treatment reduced bone loss in association with reduction in oxidative stress likely *via* activation of sirtuin-1/AMPK pathway. Although these findings are in general agreement with our findings in relation to the role of AMPK in pathogenesis of periodontitis, it is important to note that resveratrol has multiple other effects (29). Thus, our observations utilizing the AMPK KO mouse model more firmly establishes the role of AMPK in pathogenesis of periodontitis.

Another major finding of our study relates to the presence of ILCs in periodontal tissues, as revealed by electron microscopy, and their modulation by both periodontitis and AMPK. Accordingly, ILC1s, ILC2s, and ILC3s were markedly increased in ligature-induced periodontitis, and the effect was more robust for ILC2s. While AMPK knockdown did not affect frequencies of ILCs in sham control mice, induction of periodontitis further increased frequencies of ILCs but the more prominent effect on ILC2s persisted. Importantly, further *in vitro* analysis of periodontal ILCs indicates a certain level of plasticity, best observed for ILC3s, suggestive of potential role(s) for “subtypes” of ILC3s in periodontitis. This contention is based on our observation of reduced expression of CCR6 by ILC3s, under baseline and in response to treatment with IL-1β and IL-23, of periodontal tissue of periodontitis model than control animals (Figure 3C); these *in vitro* observations were more pronounced in response to treatment with cytokines. In this context, it is important to note that ILC3s play complex roles in regulation of inflammation in other conditions [e.g., inflammatory bowel disease (IBD) (30)]. Indeed, dual roles for ILC3s have been suggested depending on cytokine profile. While a protective role is attributed to IL-22, likely produced by ILC3s, in innate and adaptive IBD models, expressions of INF-γ and IL-17 are believed to drive inflammation in innate IBD models (30). Since CCR6 regulates expression of ILC3s-related cytokines (i.e., INF-γ, IL-17, and IL-22), it is likely that reduction in CCR6 expression by ILC3s of periodontitis model may reflect a compensatory mechanism to curtail inflammation by reducing potential for generation of INF-γ and IL-17 while increasing IL-22 generation, aspects that require further investigation.

In light of the more marked expansion of the pool of ILC2s in periodontitis, we also explored mRNA expression of ILC2s-related cytokines, namely IL-5 and IL-13; we also determined expression of IL-33 since it is a pivotal regulator of function of ILC2s. While periodontitis primarily increased expression of IL-33, knockdown of AMPK markedly increased expression of each cytokine in periodontal tissues of mice with ligature-induced periodontitis. Given pro-inflammatory functions that are attributed to these cytokines, future studies should determine protein levels for relevant cytokines as message and protein expressions may not necessarily correlate. Nonetheless, it is intriguing that IL-33, but not IL-5 or IL-13, is increased in the WT/periodontitis group but knockdown of AMPK “unleashes” expression of ILCs-2-related cytokines. These observations raise the possibility that suppression of ILCs-2-related cytokines in WT/periodontitis group may be a compensatory mechanism to curtail inflammation. In this context, it is noteworthy that a recent report does not show upregulation of IL-5 and IL-13 in response to “low dose” IL-33 in mice suggestive that the effect of IL-33, with respect to cytokine generation by ILC2s, is dose and context-specific (31). Importantly, however, upregulation of IL-5 and IL-13 in AMPK KO/periodontitis mice further substantiates our working hypothesis that AMPK is a modulator/regulator of ILCs. Since AMPK is the target of a number of drugs, including anti-inflammatory agents such as salicylates (16), our observations raise the prospect of targeting AMPK as a means of modulating ILCs to beneficially affect conditions where immune and inflammatory responses play major pathogenic roles including periodontitis.

In light of our observations with the murine model of periodontitis, we also explored the relevance of our findings to human periodontitis. Overall, our observations indicate remarkable similarities between the murine model of the disease and the human condition as reflected by more marked increase in ILC2s and increased mRNA expression for IL-33 and IL-5. Thus, while the pattern of changes in ILCs was similar between the murine and human periodontitis, cytokine expression profile was intermediate between the two conditions. Clearly, aside from species differences, a number of other variables (e.g., stimulus for disease induction, progression, duration, and severity) should temper far-reaching conclusions. Nonetheless, as supported by other studies (18), our collective observations suggest that the ligature-induced murine model of periodontitis is a reasonable animal model to investigate pathogenic mechanisms.

In conclusion, for the first time, we have shown the presence of ILCs and relevant cytokines in periodontal tissues in both the murine model of periodontitis and human subjects with the disease. We further show that AMPK modulates ILCs and related cytokines in this condition. Given the emerging role of ILCs in immune and inflammatory responses, unraveling of the mechanisms that regulate their function could lead to identification of novel targets of therapy to treat human periodontitis.

### Perspectives

Periodontitis is a very prevalent and progressive disease that can ultimately lead to loss of dentition. Aside from esthetic considerations and social impact on those afflicted with the disease, loss of teeth adversely impacts nutritional status thereby affecting general health of human subjects. These aspects coupled with association of periodontitis with a number of systemic diseases, including cardiovascular diseases, makes it imperative to unravel novel immune and inflammatory mechanisms underlying etiopathogenesis of periodontitis. This investigation should eventually help to identify immunomodulator(s) to prevent or curtail the progression of the disease.

For our investigation, and as described earlier, we explored whether AMPK modulates/regulates ILCs in periodontitis. Given the emerging and essential roles of ILCs in regulation of immunity and inflammation, there is intense interest to determine differential contributions of each subset of ILCs to various disorders. With respect to periodontitis, it is of interest that while the disease increased each subset of ILCs, the impact was more marked for ILC2s. ILCs demonstrate similarities with Th cells and functional properties of ILCs-2 have led to the suggestion that they represent the innate counterparts of Th2 cells. Given the more robust upregulation of ILC2s in periodontitis, it would be of interest to determine whether modulation of ILC2s can affect the development and course of the disease. Relying on the information regarding regulation of functional phenotypes of Th cells, a likely candidate would be transforming growth factor-β (TGF-β) as its signaling may contain and diminish inflammatory responses (32–34). Indeed, regulation of ILCs by TGF-β has been the subject of a recent report (35). Accordingly, it is shown that mice lacking TGF-β signaling in NKp46^+^ cells display markedly reduced numbers of salivary gland ILCs. Loss of TGF-β receptor 2 also abrogated expression of some salivary gland ILCs effector molecules. Interestingly, however, TGF-β receptor 2 deficiency had minimal impact on the phenotypes of ILC1s from liver and gut (35). These observations underlie tissue differences and complexity of regulation of ILCs. Nonetheless, our data show upregulation of IL-33 in both the murine model of periodontitis and the human condition. Since both IL-33 and TGF-β are known for context-dependent effects (32–34), it would be of interest to explore potential cross-talk between them in relation to development and differential regulation of ILCs in periodontitis (and other chronic inflammatory conditions).

## Ethics Statement

For human samples: the institutional human review board (IRB) approved the study protocol. All human samples were exempted and considered as non-human subjects due to the sampling process and clinical considerations. For animal samples: this study was carried out in accordance with the recommendations of guidelines of Institutional Animal Care and Use Committee of Augusta University. The use of animals for this study conformed to guidelines of Institutional Animal Care and Use Committee of Augusta University. The protocol was approved by the Institutional Animal Care and Use Committee of Augusta University. The animals were housed under identical standard conditions with free access to food and tap water.

## Author Contributions

XQ, NS, MH, BM, LP, LY, RE, ME, and BB: conceived, designed and performed experiments. CS and JW: provided clinical samples. MM, RA, JY, KD, and OA: provided consultation and contributed in experimental design and writing of manuscript.

## Conflict of Interest Statement

The authors declare that the research was conducted in the absence of any commercial or financial relationships that could be construed as a potential conflict of interest.
